# Effectiveness and costs of implementation strategies to reduce acid suppressive drug prescriptions: a systematic review

**DOI:** 10.1186/1472-6963-7-177

**Published:** 2007-11-05

**Authors:** Hugo M Smeets, Arno W Hoes, Niek J de Wit

**Affiliations:** 1 Julius Centrum for Health Sciences and Primary Care, UMC Utrecht, Heidelberglaan 100, 3584 CX Utrecht, The Netherlands; 2 Agis, Van Asch van Wijckstraat 55, 3811 LP, Amersfoort, The Netherlands

## Abstract

**Background:**

Evaluation of evidence for the effectiveness of implementation strategies aimed at reducing prescriptions for the use of acid suppressive drugs (ASD).

**Methods:**

A systematic review of intervention studies with a design according to research quality criteria and outcomes related to the effect of reduction of ASD medication retrieved from Medline, Embase and the Cochrane Library. Outcome measures were the strategy of intervention, quality of methodology and results of treatment to differences of ASD prescriptions and costs.

**Results:**

The intervention varied from a single passive method to multiple active interactions with GPs. Reports of study quality had shortcomings on subjects of data-analysis. Not all outcomes were calculated but if so rction of prescriptions varied from 8% up to 40% and the cost effectiveness was in some cases negative and in others positive. Few studies demonstrated good effects from the interventions to reduce ASD.

**Conclusion:**

Poor quality of some studies is limiting the evidence for effective interventions. Also it is difficult to compare cost-effectiveness between studies. However, RCT studies demonstrate that active interventions are required to reduce ASD volume. Larger multi-intervention studies are necessary to evaluate the most successful intervention instruments.

## Background

Analysis of the use of dyspeptic medication demonstrate that acid-suppressive drugs (ASD) are prescribed in 10% of the population each year [1-3]. Three percent of these patients are chronic users (>180 DDD), mostly of Proton-Pump Inhibitors (PPI), of which prescriptions increase 5% every year [4,5]. Together dyspeptic patients account for almost 11% of the pharmaceutical budget of the public insurance companies in the Netherlands. The Dutch multi-disciplinary guideline on 'Dyspepsia management' recommends PPI therapy for typical reflux symptoms for a maximum of eight weeks [6]. Only severe oesophagitis grade C/D requires long-term treatment with PPI. In most other cases gradual termination is possible.

However, many physicians repeat prescriptions without systematic evaluation of symptoms. Consequently Dutch national prevalence data of oesophagitis do not match with the rate of people using ASD, indicating that prescription recommendations are not adequately implemented [7-9].

Many patients with recurrent dyspeptic or reflux complaints also believe they have to use ASD lifelong [9-13]. Rebound effects and not explicit placebo-effects are additional factors for patients' pressure for medication. This calls for GP assisting interventions, aiming at cessation of chronic ASD use [14-16]. While ASD users consume an increasing part of the pharmaceutical budget, more effective use of resources of health care is necessary [17,18]. Better affordable strategies to reduce ASD are required to stimulate a rational pharmacotherapy.

Traditional strategies of implementation of guidelines, like passive education or economic measurements to optimise particular prescribing management have proven to be ineffective [19-21]. Grol et al demonstrated that GPs need several attributes to comply with recommendations in guidelines [22-24]. Suggesting that interventions based on multiple strategies will be more successful when actively implemented. Earlier reviews of dyspepsia guidelines enclosed only studies with single intervention strategies of various backgrounds. Recent reviews also evaluated combined strategies like audit, feedback or outreach visits, but the information provided did not permit conclusions pertaining the effect on prescription management only [25-28]. In this review we systematically evaluate the effectiveness of intervention methods for implementation of dyspepsia guidelines with the objective to reduce the volume and costs of ASD prescriptions.

## Methods

We performed a systematic literature search from 1995 to 2004 in Medline, Embase and the Cochrane Library which included search parameters of the following subject heading terms: 'dyspepsia', 'guideline', 'medication', 'implementation' and 'costs effectiveness'. Furthermore reference sections in original papers and reviews were screened to find studies otherwise published.

Selection of studies was done in two stages. In the first stage studies were screened by titles and abstracts for the description of involvement in an intervention aimed at changing management of dyspepsia. In the second stage the full text of the selected articles was retrieved to proceed the final selection by two criteria. The first inclusion criterion was that studies met criteria for adequate methodology and design. RCT and cohort follow up studies demonstrating quality criteria of evidence (A, B) set by Jailwala et al, were considered eligible to be included in the review [29]. The second inclusion criterion was the presence of outcome measurements related to reduction of medication: proportion of patients that stopped; the number of prescriptions or mean dosage of ASD; effectiveness of diagnostic tests on prescriptions; prescription costs or total disease related costs. Finally we classified the differences in strategy by which guidelines were implemented in daily clinical care: passive hand-over of (education) materials (I), or active strategies by a single (II) or multiple (III) interaction with patients and/or practitioners.

The criteria selection for design and outcome assessment in the second stage was performed independently by a second reviewer (NdeW) and disagreement was resolved by consensus. Non-systematic studies that did not evaluate the intervention or did not report outcome measurements were excluded. After a description of the type of intervention of the included studies, from each study the data pertaining to the effectiveness of the intervention were extracted. Outcome figures of prescriptions volume and costs, as well as the expenditures per patient were analysed to compare the differences in effects related to the intervention method.

## Results

**Figure 1:**
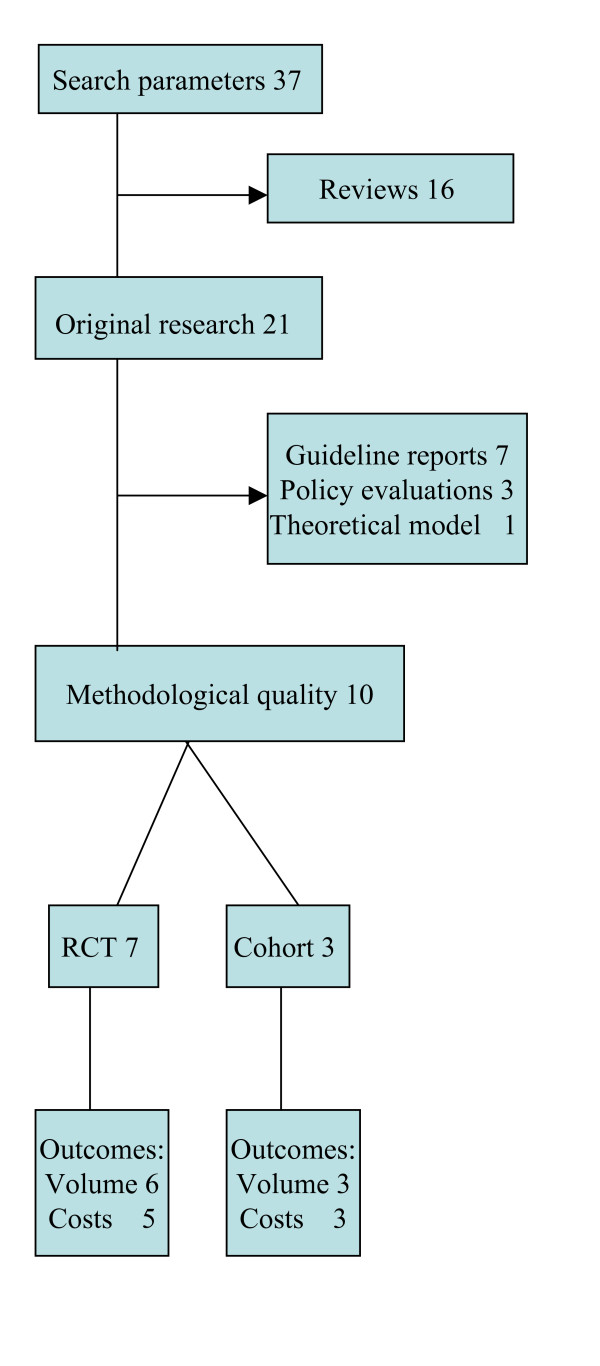
Eligible studies assessed in review.

We found 37 articles that met the inclusion criteria, 26 in the search selection and 8 in the reference sections of studies. Of these articles, 16 were marked as review articles and 21 represented original research reports (Figure 1).

Seven of the original studies were excluded because they described guidelines or measured the compliance to guidelines without reporting relevant outcomes [30-36]. Another three studies were excluded because they were limited to policy evaluations and one study was excluded because it described a theoretical model [37-40].

Finally, 10 studies were eligible to be included in analysis (Table 1) [41-50]. Seven of these studies were designed as RCT (A2) [43-48,50]. The inclusion of the research population and baseline similarity for the intervention was well described and five of them included intention to treat analysis, but most studies had shortcomings in reporting outcomes and statistics.

**Table 1:** Studies included in the Review with specifications of implementation strategies and efficacy of treatment and costs

**Authors, year**	**Research design**	**Evidence quality**	**Study characteristics: Population; i+c gr. (A) Participants (B) Stakeholders (C)**	**Method characteristics: Intervention type (A) Content guideline (B) Practical Attributes (C)**	**Implementation strategy**	**Results treatment**	**Results costs**
41. Bursey & Crowley 2000	Dynamic population cohort	B	A: 110.000 residents of NF-land, Canada B: All GPs C: Government	A: Authorisation program for reimbursement. B: Patient selection for PPI use. C: Algorithm for prescription management.	I. passive	> 80% decrease PPI	PPI < 82% ($1.3 mil) first year; <62% after 2 years. ASD <36% ($2.0 mil) first year; <16% after 2 years
42. Ladabaum & Fendrick 2001	Prospective multicentre trial.	B	A: P. ulcer patients (54+39) B: PC-centres (3+3), GPs? C: University Michigan.	A: Interactive sessions by GE. B: Test & treat strategy C: H. pylori serological test for PC.	III. multiple	32% more tests; same referrals; 31% less prescriptions (p > .001)	79% in intervention group ($ 122 pp) (p = .17)
43. Chan & Patel 2001	RCT	A2	A: All dyspepsia patients B: GPs (133+146); voluntary Hampshire C: Health authority	A: Posted guidelines and reinforcement visits by NP B: Management dyspepsia, H pylori. C: Wall chart, booklet	III. multiple	-	5% decrease in medication
44. Huren-kamp & Grund-meijer 2001	RCT	A2	A: H. pylori patients (89/85) B: 48 GP practices, voluntary C: University Amsterdam	A: Education of protocol; support by NP. B: Tapering prescriptions of ASD by doses and on demand treatment. C: follow up patients by NP.	III. multiple	Decrease of 1,5 PDD; 40% stopped ASD (ns); More HP neg, more H2RA	-
45. Weynen & de Wit 2002	RCT Cluster	A2	A: 260 (99/73/88) patients B: 28 GPs; voluntary C: University Utrecht	A: Education program, financial incentives and personal feedback. B: H. Pylori diagnosis and treatment C: Dyspepsia questionnaire, HP test.	III. multiple	17% better follow-up (ns), in incentive group	Less overall costs (€46 pp; ns) in incentive group
46. Banait & Sibbald 2003	RCT cluster,	A2	A: Practice population (265.000) B: GP practices (57+56); voluntary NW England. C: University, GE, Health authorities.	A: Posted guidelines with education outreach and follow-up visit. B: Clinical strategies for referral. C: Open access to endo-scopies and serological tests	III. multiple	14% more referrals, 4 more tests/practice	6% more costs ASD
47. Jones & Lydeard 1993	RCT,	A2	A: Practice population (500.000) B: GPs (78+101); voluntary; Southampton C: Consensus group GP+GE	A: Consensus meetings GP-SP. B: Investigation and refer dyspepsia; appropriate use of guidelines. C: reference cards,	II. single	No difference in referrals and endoscopies	22% more prescribing costs
48. Allison & Hurley 2003	RCT	A2	A: ASD patients (321+329). B: Physicians from study C: HMO California	A: Test & treatment random group. B: T&T protocol C: Detailed instructions	II single	Less ulcerlike symptoms and abdominal pain; 8% less users' medication	Higher costs because of HP treatment (not hospital)
49. Kearney & Liu 2004	Follow up Cohort	B	A: ASD patients (432) B: GE from study C: MHO Seattle	A: Patient Interview and HP test B: Hospital stopped ASD medicine C: Instruction for GPs' review.	I. passive	71% ulcer; 29% dyspepsia; number stopped?	Hospital $34 less pp; Medication ns; Only ulcer cases
50. Krol & Wensing 2004	RTC cluster	A2	A: ASD patients (63+50) B: 20 GP practices voluntary C: University Utrecht	A: Direct mail to patients to reduce ASD. B: Postal instructions for patients. C: Instruction and flowchart	II single	17% reduction (10% stopped); no change in symptoms and quality	-

In the cohort studies (B) the number of involved GPs was unknown and outcomes were not reported sequentially and in the multi-centre study (B) was the randomisation procedure unclear [41,42,49]. None of the B qualified studies described physicians' involvement in therapy management and one of them was a direct intervention from the government [41].

Three of the 10 selected studies did not evaluate cost-effectiveness. One study focused merely on costs. Excluding the evaluation of governments' intervention on population level (unknown numbers of GPs and patients), the studies involved a total of 847 GPs and/or 3512 patients.

### Intervention methods

Intervention methods used in these studies were classified according to three pre-defined strategies. The first category focused on passive intervention without further implementation activities on individual patient or doctors level and enclosed the two cohort studies(I). The first evaluated the follow up of governmental directives [41]. The second followed up the revised GP therapy after discontinuing hospital treatment [49].

The RCTs were included in the two categories of active single (II) and active multiple (III) interventions. In the first category two studies reported effects of guidelines introduced to the physicians by education and consensus meetings and one study reported effects of a guideline addressed to patients to reduce themselves medication [47,48,50]. In the multiple active category most RCTs reported about GPs who were educated about guidelines together with active support, feedback or peer visitations during the intervention [42-46].

The selection process of practitioners and adherence to the intervention were not clearly reported in all RCT studies. The duration of the intervention period as well as the compliance in the groups was not always described systematically. In general RCTs, reported detailed about the intervention strategy.

### Effect on the number of Prescription and Diagnostic tests

The study on the authorisation program of de government caused a decrease of 80% of all PPI prescriptions [41]. The study in which was addressed to patients to reduce their medication use, ASD prescriptions decreased by 17% [50]. In the remaining eight GP centred intervention studies, half of them introduced and promoted H. pylori tests, while the other half focused on guideline education with feedback strategies, the effects demonstrated variable outcomes. In the H. pylori tests group GPs were reinstructed for treatment, or were activated for using more H. pylori tests, which resulted in two unknown numbers of less ASD users and in one number of 31% patients that ceased ASD use [49,46,42]. The use of more tests caused in one study more endoscopic referrals. The forth study of this group invited patient for a H. pylori test, which resulted in 8% less ASD users.

In the guideline and feedback group GPs held consensus meetings or were given feedback about their prescription policies, but in three of these studies effects on ASD use were not reported [47,43,45]. In the fourth study of this group GPs got a prescription protocol and were extensively visited, what resulted in 40% less ASD users [44].

In general reduction of ASD was not described systematically, but if so studies with multiple active interventions demonstrated better results.

### Costs effects

From the cohort studies (I) the authorisation program in Canada reported 62% sustained decrease of costs for PPI [41]. The other cohort study and the one with serological H. pylori tests demonstrated no significant cost effects or did not report calculations [42,49].

In the studies with single intervention method (II) the promotion of the dyspepsia protocol resulted in an increase of 22% ASD costs [47]. The other two studies demonstrated by involving patients successful reduction of ASD, but costs effect calculations were not presented [48,50]. From the multiple intervention methods (III) three studies demonstrated costs effects varying respectively of 6% increase of ASD costs, no significant changes of overall medication costs until to 5% decrease in medication costs [46,45,43].

Calculation of costs in all studies was not done systematically nor uniform. Sometimes percentages of ASD costs were given, sometimes the absolute costs and sometimes none of the two. Some studies reported overall medical costs, including ASD costs. The different costs calculations are therefore difficult to compare in this review.

## Discussion

There is no doubt that radical changes can be reached from a governments' intervention, but when doctors receive a passive mailing or recommendations of key players to pay more attention to prescription protocols the effects on the number of prescriptions and costs are disappointing [21]. Studies with more active intervention strategies that support the doctors with recommendations and visitations had better results.

In studies as identified for this review many of the interventions were concerned with eradication of H. pylori, which resulted in small changes of the number of ASD users or prescriptions. Focussing on positive effects of ASD reduction and related costs two of the three single intervention studies (II) demonstrated positive results. Among the four multiple intervention studies (III) three reported a positive cost reducing effect. On average this implies only a modest effect, comparable to the small effects observed in the earlier Cochrane reviews on the effects of changes in professional behaviour [26-28].

There was only one study that particularly intervened on the gradual termination of unnecessary use of ASD [14]. The GPs were recommended to accompany their patients which resulted in a larger decrease of ASD volumes. However costs were not calculated.

The overall conclusion is that the number of high-quality studies on effective interventions for ASD reduction is limited. In addition the incomparable methods applied to calculate prescription rates and costs preclude identification of the most effective intervention strategies. The latter can only be achieved after several studies, including similar outcome measurements evaluating distinct well-defined interventions, have become available.

Only a few quantitative studies evaluated with varying success that ASD reduction requires active intervention strategies with practical instrument for the GP. Grol and Grimshaw already showed the performance phenomenon that actual changes in practice depend on helpful attributes and in particular on attributes that will overcome identified barriers against changing behaviour [17,18]. More RCT studies on population level have to calculate more thoroughly the effects that appoint to the particular successful instruments.

Doctors need rational arguments to get cooperation from patients. As long as side-effects of long term use of ASD are unknown and rebound effects make patients afraid to stop, to negotiate with patients to reduce medication is not an attractive alternative [12,24].

From this point of view government's interventions like in Canada, which forces cooperation of patients and doctors by financial incentives, seems attractive, but sustainability is questionable [38]. An alternative could be that insurance companies introduce financial rewards, either for the doctor or the patient, to defeat barriers and enforce their negotiations. They possible too could facilitate to combine interventions, both practical instruments and financial compensation, into an effective intervention program of ASD reduction. Evaluation of these multi-interventions have to demonstrate which combinations of instruments fits GPs best.

## Conclusion

Studies demonstrate that evidence for effective interventions is limited and cost-effectiveness is often difficult to compare. Larger multi-intervention studies with similar outcome measurements and distinct interventions are needed to evaluate the most successful instruments.

## Competing interests

The author(s) declare that they have no competing interests.

## Authors' contributions

HMS conceived of the study, conducted the analysis and drafted the manuscript. NJdeW participated in the design and analysis and helped to draft the manuscript. AWH helped to draft the manuscript and approved the final concept. They all approved the final manuscript.

## Pre-publication history

The pre-publication history for this paper can be accessed here:


